# *Saccharomyces boulardii* CNCM I-745 Improves Intestinal Enzyme Function: A Trophic Effects Review

**DOI:** 10.1177/1179552217752679

**Published:** 2018-02-09

**Authors:** Margret I Moré, Yvan Vandenplas

**Affiliations:** 1 analyze & realize GmbH, Department of Consulting and Strategic Innovation, Berlin, Germany; 2 Department of Pediatrics, Vrije Universiteit Brussel, Brussels, Belgium

**Keywords:** *Saccharomyces boulardii* CNCM I-745, trophic effects, digestive enzymes, diarrhoea, dysbiosis, brush border membrane enzymes, polyamines, prebiotic, probiotic, yeast

## Abstract

Several properties of the probiotic medicinal yeast *Saccharomyces boulardii* CNCM I-745 contribute to its efficacy to prevent or treat diarrhoea. Besides immunologic effects, pathogen-binding and anti-toxin effects, as well as positive effects on the microbiota, *S boulardii* CNCM I-745 also has pronounced effects on digestive enzymes of the brush border membrane, known as trophic effects. The latter are the focus of this review. Literature has been reviewed after searching Medline and PMC databases. All relevant non-clinical and clinical studies are summarized. *S. boulardii* CNCM I-745 synthesizes and secretes polyamines, which have a role in cell proliferation and differentiation. The administration of polyamines or *S. boulardii* CNCM I-745 enhances the expression of intestinal digestive enzymes as well as nutrient uptake transporters. The signalling mechanisms leading to enzyme activation are not fully understood. However, polyamines have direct nucleic acid–binding capacity with regulatory impact. *S. boulardii* CNCM I-745 induces signalling via the mitogen-activated protein kinase pathway. In addition, effects on the phosphatidylinositol-3 kinase (PI3K) pathway have been reported. As an additional direct effect, *S. boulardii* CNCM I-745 secretes certain enzymes, which enhance nutrient acquisition for the yeast and the host. The increased availability of digestive enzymes seems to be one of the mechanisms by which *S. boulardii* CNCM I-745 counteracts diarrhoea; however, also people with certain enzyme deficiencies may profit from its administration. More studies are needed to fully understand the mechanisms of trophic activation by the probiotic yeast.

## Introduction

### Saccharomyces boulardii CNCM I-745

Numerous clinical studies, almost all performed with lyophilized *Saccharomyces boulardii* CNCM I-745, also known as *Saccharomyces cerevisiae* HANSEN CBS 5926, demonstrate that the probiotic yeast is efficient and safe (*S. boulardii* CNCM I-745 is approved as medicinal drug, please refer the local Summary of Product Characteristics) for the treatment and prevention of diarrhoea of various causes.^1–6^ *S. boulardii* CNCM I-745 has also been used in a variety of different clinical conditions, including human immunodeficiency virus^7^ and *Helicobacter pylori* infection.^8,9^ Due to its multiple mechanisms of action, positive effects in a variety of different disease settings are plausible.

*S. boulardii* CNCM I-745 has an optimal growth temperature around 37°C and a relatively high acid tolerance,^10,11^ resulting in a good survival after gastric passage. A spectrum of favourable effects, including prebiotic effects,^12,13^ toxin degradation effects,^14–17^ pathogen binding effects,^18–20^ anti-secretory effects,^21^ physical barrier effects,^22,23^ effects on the microbiota,^24–26^ and on the immune system^27^ reduces the risk to develop diarrhoea or counteract diarrhoea.

Prebiotic effects are accomplished by cell wall components of *S. boulardii* CNCM I-745, consisting of glucans, mannoproteins, and chitin which serve short-chain fatty acid producing bacteria as suitable substrates for fermentation.^12,13^ Also, *S. boulardii* CNCM I-745 restores intestinal barrier integrity by regulation of E-cadherin recycling.^20^ In several animal and human studies, a protective and stabilizing effect on the intestinal microbiota has been demonstrated, including the prevention of antibiotic-associated diarrhoea by decreasing the antibiotic-induced reduction in the intestinal microbiota, as well as by supporting a faster regeneration of the intestinal microbiota following antibiotic therapy.^24,25^

*S. boulardii* CNCM I-745 influences the infection-induced signalling cascades of its human host as well as the innate and adaptive immune system. In a healthy host, *S. boulardii* CNCM I-745 leads to a general unspecific immune system activation,^28,29^ which can be considered advantageous in preventing diarrhoea. During diarrhoea, it attenuates an over-reacting inflammatory immune response and diarrhoeic leakage of fluids into the intestinal lumen.^27^

The combined effects by the yeast reduce colonization by pathogens and preservation of the integrity of the intestinal epithelial cell layer.

This review focuses on the effects of *S. boulardii* CNCM I-745 on digestive enzymes – also known as trophic effects.^30,31^ Mainly non-clinical studies, but also a few clinical studies, have evaluated the effects of *S. boulardii* CNCM I-745 on the brush border membrane (BBM) and its intestinal enzymatic activity – we have provided a summary and have attempted to explain the involved mechanisms. The sites of action of *S. boulardii* CNCM I-745 for its multiple effects are mainly the small intestine (e.g. for trophic effects, immune effects) and the colon (e.g. for effects on the microbiota, prebiotic effects, and immune effects) – with a continuing oral supply of yeast as prerequisite.

### Search method and studies included in this review

The research was conducted in the databases Medline (http://www.ncbi.nlm.nih.gov/pubmed) and PMC (http://www.ncbi.nlm.nih.gov/pmc/), searching for ‘(boulardii) AND (trophic OR enzymes)’ (61 entries in Medline and 453 entries in PMC as of November 2017). Further literature was found searching for terms relevant to the specific topic (e.g. combinations of brush border, polyamine, probiotic, and prebiotic) and following-up literature citations.

### The intestinal BBM

The BBM is the site of terminal carbohydrate digestion as well as nutrient and water absorption. Its microvilli-covered surface causes a substantial increase in surface area as opposed to flat epithelia. The BBM harbours digestive enzymes, as well as transporters that allow absorption of the digested nutrients.^32,33^

During diarrhoeal episodes (e.g. due to viral infections and/or inflammation), intestinal epithelial cells are damaged or killed. If they become replaced with immature cells, these are initially deficient in brush border enzymes and transporters necessary for absorption of water and nutrients. As a consequence, osmotic effects due to nutrient malabsorption, shifting even more water to the intestinal lumen, make the diarrhoeal episodes more severe.^34–36^ Thus, therapies to protect intestinal epithelial cells or at least to improve their enzymatic turnover will help to counteract or prevent diarrhoea.

Conversely, a decrease in enzymes involved in the digestion of nutrients – especially lactase – is frequently observed in acute and chronic enteropathies.^37,38^ The inherited sucrase-isomaltase (SI) deficiency leads to sucrose malabsorption causing diarrhoea and abdominal cramps.^39^ Accordingly, it should generally be advantageous to increase the concentration of digestive enzymes within the BBM (measured as an increase in enzyme activity per gram of total protein) – as well as the rate of nutrient uptake, to counteract diarrhoea.

## Effects of ***S. boulardii*** CNCM I-745–Secreted Enzymes

*S. boulardii* CNCM I-745 was found to secrete a number of different digestive enzymes. Although such enzyme activities most certainly are a strategy of the yeast to metabolize proteins and peptides for its own purposes, these activities could be considered advantageous within the human intestinal tract – especially under conditions like intestinal infection and/or inflammation, or for individuals lacking certain enzyme activities, or in an immature intestine.^40^

### Effects of S. boulardii CNCM I-745 on saccharide digestion

*S. boulardii* CNCM I-745 is known to secrete a highly active sucrase (more than 8000 units/g protein).^41^ Accordingly, *S. boulardii* CNCM I-745 has been used in the treatment of children who had diarrhoea due to the congenital deficiency for SI.^39^ The application of *S. boulardii* CNCM I-745 resulted in 70% reduced breath hydrogen (derived from non-digested carbohydrates fermented in the colon), in parallel with a complete loss or clear reduction in clinical symptoms.^39^ Likewise, a *S. cerevisiae*-derived preparation, sacrosidase, with 6000 IU of sucrase activity per microgram of protein, was beneficial in patients with congenital SI deficiency.^42^

More recently, *S. boulardii* CNCM I-745 was used in 21 volunteers on miglustat therapy in a double-blind, placebo-controlled, cross-over study. The glucose analogue miglustat, which is used to treat rare metabolic diseases (type 1 Gaucher disease and Niemann-Pick disease type C), is known to inhibit intestinal disaccharidases, mainly SI. The mean number of diarrhoea days was lower with miglustat + *S. boulardii* CNCM I-745 (0.8 [SD: 2.4] days) than with miglustat plus placebo (1.3 [SD: 2.4] days), as a statistical trend (no significance) in favour of the *S. boulardii* CNCM I-745 treatment.^43^

Also, other yeast-derived saccharidase activities have been found: treatment of growing rats with *S. boulardii* CNCM I-745 resulted in an increase in α,α-trehalase activities of 25% to 45% in filtered endoluminal fluid and intestinal mucosa samples compared with controls.^44^ However, *S. boulardii* CNCM I-745 does not seem to produce significant maltase, neutral lactase, or acid β-galactosidase activities on its own.^41^

### Protein and peptide hydrolysis by S. boulardii CNCM I-745

*S. boulardii* CNCM I-745 secretes a 54-kDa protease, which is capable of inactivating toxins A and B of *Peptoclostridium [Clostridium] difficile*.^14,15^ However, its specificity appears to be much broader, which can be derived from the fact that various ileal brush border proteins were degraded when exposed to *S. boulardii* CNCM I-745-conditioned medium,^16^ and that the protein methaemoglobin can also serve as enzymatic substrate.^15^ In a study on suckling rats, the oral treatment with *S. boulardii* CNCM I-745 significantly enhanced jejunal and ileal mucosal leucine-aminopeptidase activities. As cause for this enhanced activity in peptide digestion, the authors found that *S. boulardii* CNCM I-745 releases a leucine-aminopeptidase, a zinc-binding metalloprotease with 108 and 87-kDa subunits belonging to the M1 family of peptidases.^40^

Both yeast protease activities have evolved towards the digestive benefit of the yeast. However, they can be considered advantageous in enhancing human digestion of proteins and peptides, which is, within the intestine, normally achieved by the intestinal *N*-aminopeptidase (aminopeptidase *N*; APN).

This enzyme was found to be induced in the BBM of small bowel-resected rats treated with *S. boulardii* CNCM I-745^45^ (also see below) – thus, the yeast enhances protein and peptide hydrolysis by multiple mechanisms.

### Alkaline phosphatase of S. boulardii CNCM I-745

*S. boulardii* CNCM I-745 secretes an alkaline phosphatase.^17^ This enzyme is capable of inactivating *Escherichia coli* lipopolysaccharide by dephosphorylation. However, it can also dephosphorylate a number of other substrates. For *S. boulardii* CNCM I-745, this activity may be important to generate phosphate, as well as the moieties to which the phosphate had been bound. Within the intestinal tract, the capacity of *S. boulardii* CNCM I-745 not only makes phosphate available but also inactivates toxins and reduces inflammatory signals. This adds to the similar activity of the intestinal alkaline phosphatase (IAP).^46^

## Effects of ***S. boulardii*** CNCM I-745 on BBM Enzymes and Nutrient Transporters

### Effects of S. boulardii CNCM I-745 in healthy human or rats

When *S. boulardii* CNCM I-745 was administered orally to healthy rats or humans, no morphological alterations of the BBM could be found.^41,45,46^ Also, no increase in mucosal mass could be detected.^29^ However, a slight but significant increase in the mucosal DNA content of the jejunum and ileum in response to *S. boulardii* CNCM I-745 treatment was observed, possibly due to the exogenous supply of DNA provided by the yeast itself. In addition, *S. boulardii* CNCM I-745 leads to an increased intestinal secretion of the secretory component of immunoglobulins and secretory immunoglobulin A (IgA),^29^ an effect that may improve defence against pathogens.

Several studies provide evidence that the oral administration of *S. boulardii* CNCM I-745 exerts trophic effects on the mucosa of the small intestine. Already, in 1986, Buts et al^41^ reported a significant increase in the specific activities of sucrase (corresponding enzyme: SI), lactase (corresponding enzyme: lactase-phlorizin hydrolase, LPH), and maltase (corresponding enzyme: maltase-glucoamylase, MGA) in the BBM of biopsies from human volunteers who had ingested 1000 mg *S. boulardii* CNCM I-745 per day for 2 weeks. Similar results were obtained when treating rats with *S. boulardii* CNCM I-745, even if the yeast was heat killed before treatment.^41^

Ten years later, Jahn et al^46^ showed similar results by demonstrating an increase in lactase, α-glucosidase (corresponding enzyme: MGA), and IAP activity in the BBM of duodenal biopsies of human volunteers receiving *S. boulardii* CNCM I-745, using an in situ enzyme activity assay within tissue sections. The enzyme activities were comparable both for basal and apical parts of the BBM villi.^46^ Intestinal alkaline phosphatase has a key function in dephosphorylation of pro-inflammatory bacterial moieties, including lipopolysaccharides, unmethylated cytosine-guanosine dinucleotides, and flagellin as well as extracellular nucleotides such as uridine diphosphate.^47^ Dephosphorylation of lipopolysaccharides from the cell wall of gram-negative bacteria prevents their migration across the intestinal epithelium.^48,49^ At the same time, the enzymatic products of IAP, e.g. phosphate, are actively taken up as useful nutrients.^50^ The loss of IAP expression or function is associated with increased intestinal inflammation, dysbiosis, and bacterial translocation. Patients with inflammatory bowel disease (IBD) or coeliac disease have reduced IAP messenger RNA (mRNA) expression in inflamed tissues.^51,52^

### Effects of S. boulardii CNCM I-745 in models of short bowel syndrome

Patients with small bowel resection undergo a transitional phase with massive fluid and electrolyte loss with reduced nutrient absorption while at the same time a morphometric and functional compensatory adaptive response (villus hyperplasia) is taking place.^53^

In a rat model, 60% proximal small bowel resection resulted in mucosal hyperplasia with significant decreases in the specific and total activities of sucrase, lactase, and maltase. *S. boulardii* CNCM I-745 had no effect on mucosal hyperplasia but upgraded the specific disaccharidase activities to the level of control rats, which were only transected.^54^

Besides inducing digestive enzymes, *S. boulardii* CNCM I-745 also caused an increase in the sodium dependent d-glucose uptake in the BBM of rats with a 60% proximally resected small intestine, measured in BBM vesicles as a function of time and glucose concentration in the incubation medium.^54^ In agreement with this, the BBM of resected rats treated with *S. boulardii* CNCM I-745 was found to have an enhanced expression of the sodium glucose cotransporter 1 (SGLT1) when compared with resected controls.^54^

Increased activities of sucrase, glucoamylase, and APN were observed in the BBM (mucosal samples) of small bowel-resected rats (50% mid-jejunoileal resection) treated with *S. boulardii* CNCM I-745.^45^ However, in an 80% intestinal resection model with young adult rats, *S. boulardii* CNCM I-745 (2/3 of the dose used in most other studies) did not seem to be helpful in augmenting gut adaptation.^55^

### Effects of S. boulardii CNCM I-745 in broiler chicken

A recent study examined the trophic effects of *S. boulardii* CNCM I-745 administration in broiler chicken in comparison with a chicken on a diet supplemented with the antibiotic virginiamycin. The yeast group had higher adenosine triphosphatase, γ-glutamyl transpeptidase, lipase, and trypsin activities, however, no significant improvement in amylase activities.

Also, the *S. boulardii* CNCM I-745 group had increased villus height, width, and number of goblet cells, as well as increased numbers of IgA-positive cells and increased production levels of tumour necrosis factor α, interleukin 10, transforming growth factor β, and secretory IgA.^56^ Although the comparison group was treated with an antibiotic (instead of no treatment), a general digestive enzyme and digestive tract stimulating effect along with a boost of the immune system can be postulated.

## Trophic Effects of ***S. boulardii*** CNCM I-745 – The Role of Polyamines

### Polyamines in the intestinal tract

The biogenic polyamines spermine and spermidine are ubiquitous in all living organisms and implicated in many biological processes, such as cellular growth, memory performance, and metabolism.^57–59^ Spermidine is enzymatically formed from putrescine and is a precursor to spermine formation.^60^ The intracellular polyamine content has a positive correlation with the growth potential of a cell.^61^ Thus, in cancer cells, upregulation of polyamine biosynthesis may be the consequence rather than a cause of this pathology.^62^

Increased polyamine degradation may have a role in the development of diseases.^63^ A perturbed pattern or a decreased level of polyamines has been reported in neurodegenerative disorders^64,65^ and with ageing.^66^ Counteracting this effect, spermidine was neuroprotective, e.g. in a model of Huntington disease,^67^ age-induced memory impairment,^68^ or Parkinson disease.^69^

Increased polyamine levels are essential for metabolically active cells in comparison with resting cells, and stimulation of polyamine synthesis is followed by increased rates of DNA, RNA, and protein synthesis.^70,71^ Within cells, most polyamines can be found in a polyamine-RNA complex (in bovine lymphocytes, 57%), influencing the RNA structure.^55^ However, there are also certain amounts of polyamines bound to DNA (13%), ATP-Mg^2+^ (12%), and phospholipids (3%), leaving only a small fraction of free polyamines (15%).^72^ Polyamines lead to improved ribosome assembly and protein synthesis for many different growth-related mRNAs. Also, polyamine-mediated modulation of transcription (B to Z conversion of certain DNA sequences; influencing DNA condensation) has been reported.^72^

A direct influence on phosphorylation of kinases can be explained by the polyamine affinity to ATP-Mg^2+^.^73^ Furthermore, polyamines can interact with ion channels (e.g. inwardly rectifying potassium channels) and control their activity, and influence cell cycle regulation and support of membrane function.^74^ In the post-resectional rat model, the adaptive mucosal hyperplasia of the small intestine can be abolished by inhibiting ornithine decarboxylase (ODC) activity.^75^ ODC is essential for polyamine synthesis. Thus, in such a model, enteral and intravenous putrescine or spermine restores adaptive growth,^76^ so do precursors such as ornithine α-ketoglutarate.^77^ If diamine oxidase (DAO), the enzyme responsible for the breakdown of polyamines, is inhibited, this also enhances the proliferative response due to the elevated polyamine levels.^78^

Lyophilized *S. boulardii* CNCM I-745 contains a measurable content of the biogenic polyamines spermine, spermidine, and putrescine. The administration of *S. boulardii* CNCM I-745 to suckling and weanling rats significantly increased the spermine and spermidine levels within the jejunal mucosa.^79^

Interestingly, an increase in sucrase and maltase activity could not only be observed in rats in response to administered *S. boulardii* CNCM I-745^41^ but also in suckling and weanling rats when supplying polyamines, e.g. spermidine instead of *S. boulardii* CNCM I-745. In a rat model with a 60% proximal small bowel resection, an increase in mucosal polyamine concentrations induced by *S. boulardii* CNCM I-745 was observed.^54^

Thus, it is plausible that polyamines and *S. boulardii* CNCM I-745 can enhance the expression of intestinal enzymes, and that the polyamines supplied by *S. boulardii* CNCM I-745 are the signal which induces an increased expression of digestive enzymes and nutrient transporters, along with a variety of other cellular changes due to a general activation of cell proliferation and differentiation.

In rats with proximal enterectomy, treatment with *S. boulardii* CNCM I-745 did not only result in increased mucosal polyamine concentrations but was also additionally associated with a significant increase in DAO activity.^54^ This enzyme, which degrades histamine as well as polyamines, is released from the intestinal mucosa via vesicles and carried to the circulation by the lymphatics.^80^ Such negative feedback loop is plausible to control polyamine levels.

### Polyamine regulation within the yeast

Oxidative stress in yeast causes induction of antioxidant proteins (heat shock proteins, superoxide dismutase) and a G2 cell cycle arrest of variable duration (allowing more time for DNA repair). In a study, this duration could be increased by spermidine and spermine export, as well as by adding spermine extracellularly to a yeast mutant deficient in spermine export.^81^ At the same time, it is known that spermidine and spermine are able to scavenge free radicals.^82^ Thus, during oxidative stress, the presence of spermine and spermidine in the exterior surrounding the yeast cell can be considered advantageous for cellular protection while at the same time exhibiting signalling function.^81^ As a hypothesis, the acidic pH in the stomach as well as the intestinal environment will also cause stress for the yeast cells, which may enhance their spermidine secretion.

When intracellular polyamine was depleted in yeast, this resulted in a shortened chronological life span and evoked markers of oxidative stress and necrosis. Interestingly, it was found that extracellular spermidine treatment extended the life span in yeast, worms, flies, and human immune cells. At the same time, spermidine induced autophagy in yeast, worms, and flies. Autophagy is believed to be essential for healthy ageing and longevity. When the capability for autophagy was genetically abrogated, this resulted in a loss of spermidine-inducible life span extension.^83^

## ***S. boulardii*** CNCM I-745–Induced Mitogenic and Metabolic Signal Transduction

**Figure 1.**
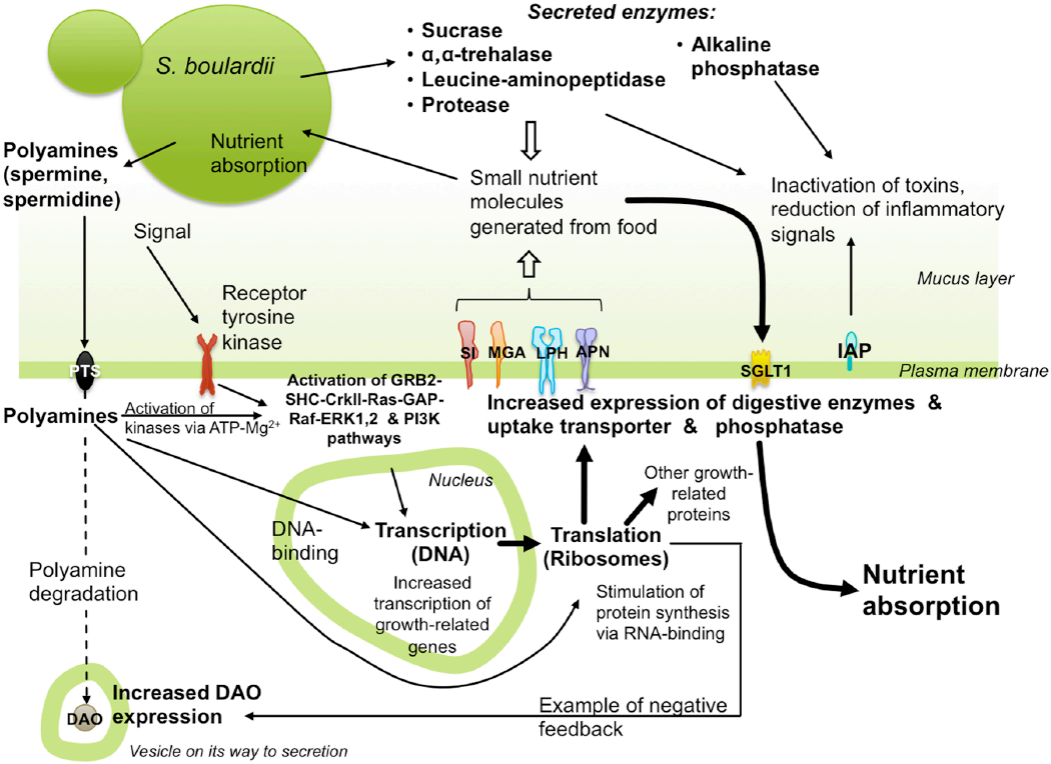
Schematic overview of the effects of *Saccharomyces boulardii* CNCM I-745 on enzymes within the small intestine. Even though not all mechanistic details are understood, the following mechanism appears plausible from the published literature: the yeast provides increased levels of polyamines, which are taken up by a polyamine transport system (PTS) and/or function as signalling molecules (other signals are also possible). As a result, translation, transcription, and kinase activities are activated, thereby inducing an increased expression of digestive enzymes, uptake transporter SGLT1 (sodium glucose cotransporter 1), and IAP (intestinal alkaline phosphatase) (likely, other targets of induction remain to be discovered). Polyamines are also generally observed to induce growth. One observed negative feedback mechanism is the increase in the polyamine degradation enzyme DAO (diamine oxidase). In addition, the yeast provides enzymes of its own, which help digest the supplied food, for the profit of both yeast and host. The combined effects will lead to an improved nutrient absorption, as well as to a faster adaptation towards a normal situation, in case that the small intestine is perturbed by disease or other causes for nutrient malabsorption. As an extra effect, the S. boulardii CNCM I-745-induced increase in alkaline phosphatase activities will inactivate toxins and reduce inflammatory signals. Abbreviations and brief explanations:
APN, aminopepedidase N (alanyl aminopeptidase, neutral brush border aminopeptidase, N-aminopeptidase) – digests peptides generated from hydrolysis of proteins by gastric and pancreatic proteases, upregulated in response to S. boulardii .^40,45^DAO, diamine oxidase – degrades histamine as well as polyamines, is released from the intestinal mucosa via vesicles, and carried to the circulation by the lymphatics.^80^ As a negative feedback, DAO is upregulated in response to S. boulardii.^54^IAP, intestinal alkaline phosphatase – dephosphorylates lipopolysaccharides derived from the cell wall of gram negative bacteria, preventing transmigration of bacteria across the epithelium; dephosphorylates other potentially pro-inflammatory ligands; upregulated in response to S. boulardii.^46^GRB2-SHC-CrkII-Ras-GAP-Raf-ERK1,2 – proteins of the MAPK pathway, including adaptor proteins SHC, CrkII, and GRB2, linking a signal receptor to a guanine nucleotide exchange factor (SOS). A signal results in an activated ERK dimer, which regulates targets in the cytosol and also translocates to the nucleus, where it phosphorylates transcription factors, which in turn regulate gene expression, most likely including genes involved in upregulation of enzyme activity. The MAPK pathway gets activated in response to S. boulardii.^84,85^LPH, lactase-phlorizin hydrolase – digestive enzyme with 2 domains, one splitting, among others, lactose, cellobiose o-nitrophenyl- β-glucopyranoside, and o-nitrophenyl- β-galactopyranoside, and the other splitting, among others, phlorizin, β-glycopyranosylceramides, and m-nitrophenyl- β-glucopyranoside.^98^ Upregulated in response to S. boulardii.^38,41,46^MGA, maltase-glucoamylase – α-glucosidase containing 2 domains with differing substrate specificity on maltose/starch and glucose oligomers with α(1→4) bonds; upregulated in response to S. boulardii.^38,41,45,46^SI, sucrase-isomaltase – α-glucosidase containing 2 domains with overlapping substrate specificity, hydrolysing oligomers with (1→6)-α-d-glucosidic linkages including sucrose; upregulated in response to S. boulardii.^41,45^SGLT1, sodium glucose cotransporter – transports glucose into enterocytes while exporting sodium; upregulated in response to S. boulardii.^54^PI3K, phosphatidylinositol-3 kinase.PTS, polyamine transport system.^99^

Altogether, there are numerous pieces of evidence that the presence of *S. boulardii* CNCM I-745 in the intestinal tract leads to an increase in digestive enzymes within the villous membrane of enterocytes. Although the signalling pathways leading to an increased enzyme activity are not completely understood, mitogen-activated protein kinase (MAPK) signalling mechanisms are very likely involved (Figure 1). In experiments using immunoprecipitation and immunoblotting of preparations from rat intestinal tissues, it was concluded that *S. boulardii* CNCM I-745 acts via the pathway GRB2-SHC-CrkII-Ras-GAP-Raf-ERK1,2.^84,85^ This MAPK pathway is known to control cellular proliferation, differentiation, and survival and may also be relevant for the upregulation of enzyme activity.

Investigations have shown that protein kinase CK2 activity is upregulated by intracellular polyamine levels.^86^ Elevated CK2 levels can be found in proliferating cells. At least in *Drosophila*, protein kinase CK2 was able to monitor intracellular polyamine levels and translate this information to modulate MAPK signaling.^87^ *S. boulardii* CNCM I-745 has also an effect on the phosphatidylinositol-3-kinase (PI3K) pathway: the phosphorylated form of p85, a critical regulatory unit of this pathway, was increased by 2.5-fold in rats treated by the probiotic.^87^

It should be considered that both the enzyme stimulatory and the anti-inflammatory activities may be regulated by overlapping signalling cascade proteins, explaining the immunologic effects of *S. boulardii* CNCM I-745. In *S. boulardii* CNCM I-745–treated rats, nuclear factor κB could not be detected, whereas a large signal was detected in controls, biochemically demonstrating the anti-inflammatory action of *S. boulardii* CNCM I-745. Also, *S. boulardii* CNCM I-745 decreased activation of p38 MAPK, a kinase responsive to pro-inflammatory cytokines and environmental stress.^84,85,88^ More studies in this area will improve the precise mechanistic understanding of digestive enzyme regulation by *S. boulardii* CNCM I-745.

## Summary and Discussion

### Trophic effects by enzyme stimulation and supply of yeast enzymes

Overall, it is apparent from the available data that *S. boulardii* CNCM I-745 is able to stimulate a number of intestinal digestive enzymes and a transporter, in growing rats, rats with shortened intestine, as well as humans. There is additional evidence of trophic effects of the yeast in broiler chicken. At the same time, *S. boulardii* CNCM I-745 supplies additional enzymes that also improve digestion.

As a limitation, the evidence is collected mostly from studies on rats, with 6 to 12 rats per group. However, a significant stimulation of the enzymes SI, LPH, and MGA, as well as IAP, has also been demonstrated within 2 pharmacologic studies on human volunteers.

Table 1 summarizes the study results of non-clinical and clinical studies regarding the influence of the administration of *S. boulardii* CNCM I-745 on the BBM and digestive enzymes. Significant trophic effects by the yeast on the stimulation of enzyme specific activity (enzymes belonging to test subjects) ranged from around 20% to 260% depending on enzyme and test system. As shown in 2 studies, an *S. boulardii* CNCM I-745–dependent increase in polyamines ranged from 20% to 160% depending on polyamine and test system. In addition, the yeast is able to supply certain enzymes, which result in up to 110% increased respective enzyme activities within subjects, again depending on enzymatic activity and test system. More and larger studies are needed to confirm the observed effects as well as their relevance in real-life settings.

**Table 1.** Non-clinical and clinical studies regarding the influence of the administration of *Saccharomyces boulardii* CNCM I-745^a^ (abbreviated with S. boulardii in this table) on the BBM and digestive enzymes.

Study	Methods and duration	Daily dosage/g of body weight^uoi^	Significant effects of *S. boulardii* (and other relevant results) *Enzyme activities: specific activities*^uoi^ BOLD TERMS: see ∆% in same order	∆% Relative size of significant trophic effects by viable *S. boulardii*^b^ (rounded)	Number/group
**Effects of *S. boulardii*/polyamines on BBM, enzymes, and nutrient transporters**					
Buts et al^41^	30-d old Wistar rats (50 g rats) 5d oral *S. boulardii* treatment versus control animals Also, control rats with heat-treated *S. boulardii*; saline control groupDuodenojejunal biopsies (peroral suction biopsy) of human volunteers before and after *S. boulardii* for 2 wk: effects on BBM morphometry and enzyme activity	1.5 mg (3× 0.5 mg)1000 mg (4× 250 mg) *per* subject	• No alteration of jejunum BBM morphology, no intracellular or BBM lesions • The yeasts located to the lumen or contacted villus cells without penetration into the epithelium or any signs of inflammation • Increase in BBM **sucrase, lactase**, and **maltase** activities above control levels both for heat-killed and viable *S. boulardii* • No change in human BBM morphology or morphometry, including villous height and crypt depth; no cellular infiltration with yeast • Increase in BBM **sucrase**, **lactase**, and **maltase** activity above basal enzyme activity (day 0)	NA NA 157%, 150%, 104%NA 82%, 77%, 75%	NA NA 6 rats/groupNA Pre-post comparison in 7 human volunteers
Buts et al^29^	Weanling Wistar rats receiving *S. boulardii* by gastric intubation from days 14 to 22 (8 d) Control groups; 0.9% saline or ovalbumin	1.5 mg (3× 0.5 mg)	• No increase in mucosal mass • A slight but significant increase in the mucosal DNA content of the ***jejunum*** and ***ileum*** in response to *S. boulardii* • Enhanced intestinal secretion of secretory component of immunoglobulins and secretory IgA in response to *S. boulardii*	NA 19%-22%, 16% NA	NA 8 rats/groupNA
Buts et al^79^	Weanling Wistar rats (20-30 d old, 100 g rats): BBM enzyme activity in response to administered *S. boulardii* (10 d from days 20-30) or administered spermine. Suckling rats^c^ also treated with spermine days 10-14	1 mg (3× 0.33 mg) by gastric intubation	• *S. boulardii* was found to contain significant amounts of polyamines (mainly spermine and spermidine): 6.79 nmol/mg lyophilized *S. boulardii* • *S. boulardii* caused increase in **spermine/spermidine** levels in the jejunal mucosa of treated rats • Induced increase in ***sucrase*** (versus controls) and ***maltase*** by *S. boulardii* and similarly by spermine (500 nmol/d/rat) in weanling rats	NA 22%, 21% 157%, 48%	NA 10 rats/group 8 rats/group
Jahn et al^46^	Duodenal biopsies (10 each) of human volunteers before and after receiving *S. boulardii* for 21 d – effects on BBM morphometry and enzyme activity: in situ technique measuring enzyme activity in biopsy sections	750 mg (3× 5 capsules of 50 mg)^d^ *per subject*	• No change in BBM morphology: no significant difference in villous surface or in crypt depth, however, trend towards increase in villous surface area • Within biopsies: increase in brush border enzyme activity of **lactase, α-glucosidase (glucoamylase)**, and **alkaline phosphatase** (basal and *apical* villi with comparable enzyme activities)	NA 20%, 50%, 24%	NA Pre-post comparison in 12 human volunteers
Buts et al^84^	Litters of growing Wistar rats, days 30-34 treated with *S. boulardii* or saline for 4 d – immunoprecipitation and immunoblotting of intestinal tissue preparations Additional experiment: Wistar rats, days 30-34, treated with 2 µg/g body weight/d of PD098059, (inhibitor of MAPKK and of ERK1,2) for 4 d, 1 h before the administration of *S. boulardii* Controls received the vehicle of the inhibitor	0.05 mg (‘50 µg within 2 doses per day’) (*Possibly 0.5* *mg-unit conversion error?*)	• Generated stimuli transduced via the following pathway: GRB2-**SHC**-**CrkII**-Ras-GAP-Raf-**ERK1,2**. Each of these signalling substrates were increased in mucosal extracts of *S. boulardii*-treated rats compared with controls • *S. boulardii* resulted in an increase in phosphorylation of ***p85***, the critical regulatory unit of the Pl-3 kinase pathway, compared with controls • *S. boulardii* induced decreases p38 MAP kinase (−23%) and NF-κB (−93%), 2 initiators of inflammation and pro-apoptotic transcription • *S. boulardii* induced decreases p38 MAP kinase (−23%) and NF-κB (−93%), 2 initiators of inflammation and pro-apoptotic transcription	485%/62%/58%^e^, 63%, 56% 127% NA NA	6 rats/group 6 rats/group 6 rats/group 8 rats/group
Sun et al^56^; see also, Rajput et al^89^; Rajput et al.^90^	For 72 d, broiler chicken received either a basal diet with 20 mg/kg virginiamycin or 1 × 10^8^ cfu *S. boulardii*/kg feed	Not stated	• The *S. boulardii* group had higher adenosine triphosphatase, γ-glutamyl transpeptidase, lipase, and trypsin activities • The *S. boulardii* group had increased villus height, width, and number of goblet cells • No significant improvement in amylase activities • The *S. boulardii* group had increased numbers of IgA-positive cells and increased production levels of tumour necrosis factor α, interleukin 10, transforming growth factor β, and secretory IgA	NA for all (control group received antibiotic, *S. boulardii* group did not)	100 broiler chicken/group, each divided into 5 replications (n = 20)
**Effects of **S. boulardii** on BBM enzymes and nutrient transporters – models of short bowel syndrome**					
Buts et al^54^	Young adult male Wistar rats (150-155 g): 60% proximal enterectomy followed by treatment of with *S. boulardii* from days 1 to 8 after surgery or saline as control Transected group as additional control	1 mg	• Proximal enterectomy induced mucosal hyperplasia with significant decreases in the specific and total activities of specific disaccharidases: *S. boulardii* had no effect on mucosal hyperplasia but upgraded mucosal **sucrase, lactase,** and **maltase** activities to the level of transected-only controls • Resected rats treated with *S. boulardii* exhibited increases in mucosal **putrescine, spermine**, and ***spermidine*** • Significant increases in **diamine oxidase activity**Increase in **sodium-dependent d-glucose uptake** by BBM vesicles in the resected group treated with *S. boulardii* • Enhanced expression of the **sodium/glucose cotransporter 1** in the BBM of resected rats treated with *S. boulardii* compared with resected controls • Enhanced expression of the ***sodium/glucose cotransporter 1*** in the BBM of transected rats treated with *S. boulardii* compared with transected controls	140%, 255%^f^, 41%^f^ 140%^f^, 14%, 14% 58% 81% 82% 243%	6 rats/group (sucrase) 8 rats/group (lactase, maltase) 12 resected; 10 (resected + *S. boulardii*) 8 rats/group 4 samples/group 4 rats/group 4 rats/group
Zaouche et al^45^	Male Sprague Dawley rats (137 ± 2 g) with 50% mid-jejunoileal resection, leaving proximal 25% of the jejunum + distal 25% of the ileum; treatment with *S. boulardii* or placebo. Also, transection controls and no surgery controls. Sacrifice after 4 and 8 d	1 mg	• No histomorphometric changes due to *S. boulardii* • After 8 d: Increased **sucrase, glucoamylase** and **N-aminopeptidase** (*total* activities only) in the jejunal remnant of small bowel-resected rats treated with *S. boulardii*, compared with resected but untreated controls • After 4 d: Increased **sucrase, glucoamylase**, and **N-aminopeptidase** (*total* and *specific* activities) in the ileal remnant of small bowel-resected rats treated with *S. boulardii*, compared with resected but untreated controls (functional adaptation)	NA Day 8 total: 95%, 47%, 115% Day 4 total: 171%, 356%, 90% Day 4 specific: 111%, 219%, 59%	NA8 rats/group except for *S. boulardii*-treated resected rats (n = 7)8 rats/group
Kollman et al^55^	Young male adult rats (150 g ± 10 g): one group with 80% intestinal resection; one group sham operated Groups further divided to receive ‘normal rat chow ± *S. boulardii* for 14 d’	0.16 mg as well as 0.6 mg	• Intestinal adaptation (increase in mucosal mass/cm) in resected animals compared with nonresected controls • No statistically significant differences between treated and untreated animals	NA	8 rats/group
**Effects of *S. boulardii*-secreted enzymes**					
Buts et al^41^	In vitro enzyme activity assay	NA	• Concentrated preparations of *S. boulardii* cells exhibited high sucrase activity and very low alkaline phosphatase activity	NA	In vitro
Pothoulakis et al^16^	In vitro: binding of [^3^H]toxin A to its brush border receptor, preincubated with *S. boulardii*-cultured suspension or filtered conditioned medium Rat ileal loops from male Wistar rats (200-250 g) pretreated with *S. boulardii* for 3 d (or control): effect of toxin A on secretion, epithelial permeability, and morphology	*Every* day for 3 d: 150 mL of 100 mg/mL *S. boulardii* suspension as drinking water → 0.44 mg/g of body weight/mL	• *S. boulardii* reduced the binding of [3H]toxin A (from *Peptoclostridium [Clostridium] difficile*) to its BBM receptor in a dose-dependent fashion • Sodium dodecyl sulphate polyacrylamide gel electrophoresis of ileal brush border exposed to *S. boulardii*-conditioned medium revealed a diminution of all brush border proteins • Treatment of rats with *S. boulardii* suspension reduced fluid secretion and mannitol permeability caused by toxin A	NA NA NA	In vitroIn vitroIn vitro
Castagliuolo et al^14^; see also, Castagliuolo et al^15^	Male Wistar rats (200-250 g) with toxin A-induced enteritis; purified human BBM and other in vitro models	Purified protease/*S. boulardii*-conditioned medium	• *S. boulardii* was found to release a serine 54-kDa protease that (also) digesed toxins A and B of *Peptoclostridium [Clostridium] difficile* and the BBM receptor of toxin A • *S. boulardii*-conditioned medium lowered fluid secretion (−38%) and increased mucosa permeability (−48%) observed after administration of toxin A in the rat ileum in vivo • Anti-*S. boulardii* protease IgG reversed this inhibitory effect • Anti-*S. boulardii* protease IgG prevented the action of *S. boulardii* on the ability of 3H-toxin A and 3H-toxin B to bind to human colonic BBM	NA NA NA	NA 6-10 rats/group NA
Buts et al^40^	Suckling Wistar rats^c^ oral treatment from days 11 to 14 (4 d) with *S. boulardii* or saline	0.5 mg (2× 0.25 mg)	• Enhancement of **jejunal** and **ileal** mucosal leucine-aminopeptidase activities by *S. boulardii* treatment due to release of a yeast leucine-aminopeptidase: a zinc-binding metalloprotease belonging to the M1 family of peptidases with a 108 and an 87-kDa subunit and an optimum at pH 8 • *S. boulardii*-enhanced leucine-aminopeptidase activities in **jejunal** and **ileal** fluids %	24%, 34% (text) 31%^f^, 61%^f^ (from Figures 5 and 6 of publication) 48%, 106%	9 rats/group 9 rats/group
Buts et al^17^	Growing rats Weaning Wistar rats^g^ or oral treatment with *S. boulardii* or saline from days 28 to 32 (5 d)	0.5 mg (2× 0.25 mg)	• *S. boulardii* was found to release a protein phosphatase that (also) inhibited *Escherichia coli* lipopolysaccharide by dephosphorylation • *S. boulardii* treatment in growing rats was able to increase phosphatase activity in the **ileum** (only trend in the jejunum)	NA 55%^f^	NA 6 rats/group
Buts et al^44^	Growing rats, *S. boulardii*, or saline treatment from days 15 to 20^h^ (6 d)	0.05 mg (‘50 µg within 2 doses per day’) (*Possibly 0.5* *mg-unit conversion error?*)	• *S. boulardii* treatment resulted in increases in α,α-trehalase activities in the **endoluminal fluid** and **intestinal mucosa** compared with control rats. • Total or partial α,α-trehalase deficiencies were observed in 28.5% of 144 adult/56 children subjects with diarrhoeic symptoms, indicating the possibility to treat trehalose intolerance with *S. boulardii*	30%^f^, 112%^f^ (from Figure 6 of publication) NA	6 rats/group Prevalence study
**Trophic effects in clinical settings**					
Harms et al^39^	Children with congenital sucrase-isomaltase deficiency: single administration of 2 g sucrose/kg body weight (or sucrose alone) followed by lyophilized *S. boulardii* (supplier not stated). The amount of sucrose non-digestion was measured by the sucrose hydrogen breath test	300 mg *per subject*	• In vitro, *S. boulardii* had a strong sucrase activity, a slight isomaltase activity, a low maltase activity, and virtually no lactase activity • With *S. boulardii* treatment, patients had reduced **breath hydrogen excretion** and loss or evident reduction in clinical symptoms (e.g. diarrhoea) • In vitro, *S. boulardii* sucrase activity was more inhibited by undiluted (pH 1) than by diluted (pH 2.6) gastric juice, suggesting preferential yeast administration on a full rather than an empty stomach	NA −70%; NA NA	In vitro 8 children with congenital sucrase-isomaltase deficiency: measurement first without *S. boulardii*; 1 wk later with *S. boulardii* In vitro
Remenova et al^43^	Volunteers on miglustat therapy (inhibiting mainly sucrase isomaltase; double-blind, placebo-controlled, cross-over study). 14 d miglustat 100 mg thrice a day + *S. boulardii/*placebo (random order separated by washout; *S. boulardii*/placebo treatment starting 2 d before miglustat) The mean number of diarrhoea days was measured	1000 mg (2× 500 mg) *per subject*	• Trend in favour of the *S. boulardii* treatment: reduction in the **mean number of diarrhoea days** with miglustat + *S. boulardii* (0.8 [2.4] d compared to miglustat + placebo (1.3 [2.4] d)	−39% (trend)	Cross-over: 21 volunteers on miglustat

Abbreviations: BBM, brush border membrane; IgA, immunoglobulin A; NA, not applicable.

a If not stated otherwise: *S. boulardii* CNCM I-745 weighed in its lyophilized form as supplied by the manufacturer (Biocodex, Gentilly, France); 2.9 × 10^9^ viable cells/mL or 10^10^ cfu/mL.

b From published data, recalculation from single values was attempted, if possible. Calculation examples: (1) Viable *S. boulardii* increased sucrase activity in the jejunum of 30-day-old rats, with hydrolysation of 23 µmol substrate/min/protein in controls and 59 µmol substrate/min/protein with addition of *S boulardii*: increase by 36 µmol substrate/min/protein or 157%.^41^ (2) *S. boulardii* reduced the mean number of diarrhoea days from 1.3 to 0.8 days: reduction by 0.5 days or −39%.^43^ The change is given relative to the control group: everything identical but without *S. boulardii*. The sequence corresponds to the sequence of the bold terms in the preceding column. Only significant effects are listed (*P* < .05), unless otherwise indicated (trend).

c Suckling rats have a low rat BBM aminopeptidase activity,^91^ as well as a low sucrase and maltase activity (but a high lactase activity),^92^ making the effect of *S. boulardii* more apparent.

d *S. boulardii* ‘Perenterol’ manufactured by Thiemann, Waltrop, Germany (10^9^ viable lyophilized cells/mL): identical to *S. boulardii* by Biocodex.

e SHC proteins p52/p46/p21, calculated from published numeric values.

f Numbers for calculation derived from published graphic.

g Weaning rats have a low BBM alkaline phosphatase activity.^93^

h Trehalase activity is virtually absent before weaning and starts to be induced by day 16.^44^

uoi Unless otherwise indicated.

### Polyamines and trophic effects: towards a mechanistic understanding

*S. boulardii* CNCM I-745 – most likely by secreting polyamines – is able to stimulate the expression of digestive enzymes (SI, MGA, LPH, APN, IAP) and nutrient transporters (SGLT1). The enzyme stimulation is likely to involve the GRB2-SHC-CrkII-Ras-GAP-Raf-ERK1,2 pathway and the PI3K pathway. These pathways may be activated by polyamines which are capable of influencing kinase activities and/or by an additional external signal. Polyamines are also known to stimulate protein synthesis via RNA binding and stabilization, resulting in an increase in growth-related and differentiation-related proteins, including digestive enzymes, which will be inserted into the BBM. Also, polyamines can interact with DNA, facilitating the generation of certain transcripts.

Thus, clinically, we postulate a general polyamine-triggered metabolic activation due to *S. boulardii* CNCM I-745 which will cause a faster regeneration of any damaged BBM areas.

Polyamine levels are regulated by increased polyamine degradation via DAO if the polyamine levels are high.

The recent study by Sun et al. highlights beneficial trophic effects by the yeast - also in broiler chicken, including the activation of several digestive enzymes compared with the administration of an antibiotic.^56^

### Clinical potential of S. boulardii CNCM I-745

*S. boulardii* CNCM I-745 secretes several digestive enzymes, including a highly active sucrase. The supply of additional yeast enzymes together with activation of intestinal enzymes and transporters by *S. boulardii* CNCM I-745 will increase digestive enzyme activities, nutrient digestion, and absorption. This is of special advantage when the digestive system is affected, e.g. by disease. Thus, patients with infectious or inflammatory diarrhoea will benefit from the increased enzyme activity and nutrient absorption induced by the probiotic yeast. Phosphatases additionally inactivate toxins and reduce inflammatory triggers.

The stimulation of the IAP by *S. boulardii* CNCM I-745, in combination with the yeast alkaline phosphatase, suggests treatment options in chronic inflammatory states such as IBD, coeliac disease, and obesity.

A large number of people have low or absent activities of certain digestive enzymes, either for genetic reasons, or due to enterocolopathies, or other chronic abdominal diseases. Lactose intolerance related to primary or more often secondary lactase deficiency (LPH deficiency) affects a wide number of people worldwide.^94^ However, there are also people deficient in α,α-trehalase,^44^ SI,^95^ or other α-glucosidases.^96,97^

*S. boulardii* CNCM I-745 is unique in offering a large variety of different digestion improving effects, increasing the activity of the major digestive enzymes. Administration of *S. boulardii* CNCM I-745 will alleviate symptoms of maldigestion induced by genetic or acquired enzyme deficiencies. Also, it can be concluded that patients with (e.g. virally induced) inflammatory diarrhoea will profit from the improved enzyme activity and nutrient absorption induced by the probiotic yeast, since this will counteract osmotic effects and thus lead to less watery stools. This reflects the current indication for lyophilized *S. boulardii* CNCM I-745 preparations.
